# Solitary fibrous tumor of the thigh with epithelioid features: a case report

**DOI:** 10.1186/1746-1596-2-19

**Published:** 2007-06-18

**Authors:** Miguel Martorell, Ana Pérez-Vallés, Francisco Gozalbo, Jose Angel Garcia-Garcia, Jair Gutierrez, John Gaona

**Affiliations:** 1 Department of Pathology, Hospital General Universitario. Valencia, Spain

## Abstract

**Background:**

Extrapleural Solitary Fibrous tumors (SFTs) have been increasingly reported. The retroperitoneum, deep soft tissues of proximal extremities, abdominal cavity, trunk, head and neck are the most common extraserosal locations reported. Microscopically they show a wide range of morphological features, and so the differential diagnosis is extensive. Immunohistochemically, they commonly express CD34, vimentin, bcl-2 and CD99. Epithelial membrane antigen (EMA) and smooth muscle actin (SMA) may occasionally be expressed. Epithelioid morphology in extrapleural SFT has only very occasionally been described (five cases reported), some of them with biphasic pattern and others with malignant characteristics.

**Case presentation:**

A SFT of the thigh with epithelioid areas in a 63 year old woman is reported. Microscopically the tumor showed areas hypo and hipercellular. At the periphery of the hipercellular areas there were nodules composed of epithelioid cells. Immunohistochemically both the spindle and epithelioid cells were positive for CD34, vimentin, bcl-2 and CD99. Epithelial, neural and muscular markers were negative. Molecular study was done and ruled out a synovial sarcoma.

**Conclusion:**

Ten cases of SFT of the thigh have been reported but to our knowledge this is the first case with epithelioid morphology affecting the extremities. Identification of this pattern of SFT is of importance, to avoid misdiagnosis with other more aggressive conditions in soft tissue.

## Background

Extrathoracic solitary fibrous tumors have been described at almost every anatomic location [1-3], but reports of tumors at the extremities or intramuscular tumors as well as those with malignant clinical behavior or atypical histologic features are rare [4]. Few cases of soft tissue epithelioid solitary fibrous tumor affecting mediastinum [5], orbit [6], neck [7] and ischioanal fossa [7,8] have been described. We present a case of solitary fibrous tumor of the thigh showing distinct biphasic morphology and demonstrating epithelioid differentiation.

## Case presentation

A 63 year old woman presented with a three year history of a painless growing mass in the groin and increasing pain in her left leg during the last year. Magnetic resonance imaging (MRI) showed a lesion measuring 11 × 7 × 7 cm. near the left hip affecting the quadriceps. The lesion was suggestive of a soft tissue sarcoma. Total body scan revealed no distant metastasis. Excisional biopsy was done, resulting in a low-grade fusocellular tumor with hemangiopericytic pattern.

Surgical treatment was carried out and the whole tumor was submitted for histopathological study. The patient was treated with radiotherapy (63 Gy). Control MRI, six months after radiotherapy, showed no lesions.

Grossly the tumor appeared as an encapsulated, tan-grey mass measuring 10 × 4 × 3 cm. At cut section the tumor was pseudolobulated, with small hemorrhagic foci, and yellow necrosis. No differences in color and consistence were found between lobules.

Microscopically, the tumor was composed of a proliferation of fusocelular cells with haphazardly distribution and varying degrees of stromal collagenization. The cellularity greatly varied in different areas with a predominance of hypercellular areas. Medium-sized thin-walled blood vessels in a hemangiopericytic growth pattern were observed, being more evident in hypercellular areas at the periphery of the lobules. Highly cellular spindle cell areas resembled fibrosarcoma and very occasionally multinucleated cells were seen. Mitosis were spare (≤ 2 mitosis in 10 HPF) but foci of coagulative necrosis existed.

At the periphery of the tumor and close to hipercellular areas we found three isolated and fairly well demarcated nodules each one measuring 12–15 mm, where cells adopted an epithelioid morphology with round, vesicular nuclei with micro nucleoli and abundant eosinophilic cytoplasm. These cells were mainly arranged in solid sheets but nests, pseudoglandular, or cleft patterns were also present. Focally artifactual shrinkage produced pseudovascular spaces. In these areas pleomorphism was moderate. No fusocellular cells were seen. Mitosis score was more than 10 in 10 HPF, some of them abnormal, and foci of necrosis were observed. A thin band of collagen isolated these epithelioid nodules from the fusocelular rich areas except in one of the nodules which both epithelioid and spindle areas merged in indistinct transition.

Cells from fusocelular and epithelioid areas showed the same immunophenotype expression: vimentin +, CD34 +, CD99 +, and bcl2 +, being completely negative for epithelial, neural and muscular markers. Ki67 immunolabeling was low in fusocellular areas (< 5%) and rather high (> 40%) in epithelioid ones. (All antibodies from Dako)

Polymerase chain reaction (PCR) for presence of a SYT-SSX1 or SYT-SSX2 fussion transcript [9] proved negative.

## Conclusion

Extrapleural solitary fibrous tumor, especially those at the extremities, still represent a rare entity of soft tissue neoplasms [10]. In a current literature review 11 cases located at thigh have been reported [4,2,11-14].

Histologically SFT are well circumscribed, and consist of bland spindle cells showing a wide spectrum of histological features ranging from hypercellular to myxoid or hialinized pattern-less hypocellular areas. Hemangioperycitomatous pattern is also evident mainly in hipercellular areas of tumors. Mitoses are infrequent and necrosis is not common in SFT. Some histological variants have been described as giant cell SFT fibroma and fat forming SFT [15,16]. Immunohistochemically SFT commonly expresses CD34, CD99 and bcl-2, epithelial membrane antigen (EMA), and smooth muscle actin (SMA) may occasionally be expressed. They are usually negative for S-100 protein, desmin and cytokeratins [17,18].

Although clusters of polygonal (epithelial-like) cells have been described in SFT of pleura and mediastinum [1] the term epithelioid SFT was proposed for the first time in 2003 by Marchevsky et al when reporting a mediastinal soft tissue with predominant epithelioid cells sharing histological and immunohistochemical features of SFT and adenomatoid tumor [5]. Since then, five cases of epithelioid SFT affecting orbit [6] ischioanal fossa [8,7] and neck [7] have been reported.

Microscopically, three of the cases reported showed a biphasic morphology coexisting areas of typical fusocellular SFT and areas of epithelioid appearance [6,7], one of the cases was predominantly epithelioid [5], and the other one showed only epithelioid areas[8]. In three of the cases, epithelioid areas were described having benign non-malignant appearance. [5,6,8], but the epithelioid component showed malignant appearance in the other two cases [7].

Pathological criteria of malignancy include large tumor size (more than 50 mm), infiltrative margins, high cellularity, nuclear pleomorphism, areas of tissue necrosis and increased mitotic index (more than 4 mitosis in 10 HPF) [11,3,19]. In our case malignant microscopic findings were present exclusively in the epithelioid areas, being completely absent in fusocellular, non epithelioid ones. This fact raises the possibility that this epithelioid areas could represent foci of malignant transformation in an otherwise long standing lesion (more than 3 years), as occurred in our patient.

The behavior of SFT is unpredictable [18]. The relationship between morphology and outcome is poor. Some "malignant" tumors behave benign while some morphologically "benign" lesions behave aggressively. In addition to the presence of histological criteria of malignancy, absence of sclerotic-hypocellular areas and tumor size more than 10 mm have been considered predictors of poor outcome [19].

Immunohistochemical results on epithelioid SFTs are quite contradictory. These epithelioid cells usually retain an immunophenotype of SFT such as CD34 +, vimentin +, CD99 + and bcl-2 + [5-8], except in one case reported that lost vimentin and bcl-2 expression [7]. Epithelial markers have also been positive. Three of the reported cases showed epithelioid cells staining with CK AE1-AE3 [5,7], and one case was also positive for MNF116 [7]. These markers were also positive in the fusocellular component of one of the cases reported [7]. Scattered epithelioid cells were positive for KL1 in one of the cases [8] and epithelial membrane antigen was negative in all cases.

The fusocellular component was positive for CKAE1-AE3 and MNF116 in one of the cases reported [7]. The expression of cytokeratins occurs mainly in epithelioid areas of SFTs [5,7], especially in areas with malignant appearance [7]. Two of the three CKAE1-AE3 positive cases had epithelioid component with histological characteristics of malignancy.

In our case, immunohistochemical stains demonstrated positivity of epithelioid cells with vimentin, CD34, bcl2 and CD99 and negativity for epithelial, neural and muscular markers.

The differential diagnosis of SFTs of soft tissues is extensive. Hemangiopericytoma is one of the lesions that bears a close resemblance with SFT of soft tissues (fascicular pattern, fibrosis, and vimentin, CD34, and bcl2 immunoreactivity). The hemagioperycitomatous pattern is present in a wide variety of tumors; in fact, in absence of this pericytic differentiation [20], most hemangiopericytomas, are considered morphological forms of SFT [18]. In a recent WHO classification of soft tissues [20]; SFT and hemangiopericytoma are considered a single entity with a morphological continuum among them. According to this hypothesis SFT of soft tissues is probably an underdiagnosed entity at present.

The epithelioid growth pattern contributes to further difficulties in the differential diagnosis of these tumors with other tumors having epithelioid features, such as epithelioid sarcoma, schwannoma, leiomyosarcoma, MPNST, hemangiopericytoma and synovial sarcoma among others.

The differential diagnosis with synovial sarcoma may prove very difficult, mainly if SFT express cytokeratin and EMA. Synovial sarcoma rarely expresses CD34. No molecular studies seeking a SYT-SSX1 or SYT-SSX2 fusion transcript have been performed in CK+ epithelioid SFT cases reported. In one case [7], the FISH analysis failed to provide any evidence of a split signal, consistent with the absence of the t(X;18) translocation. The expression of CD34 and the absence of a SYT-SSX1 or SYT-SSX2 fusion transcript, rules out the diagnosis of synovial sarcoma as occurred in our case.

**Figure 1:**
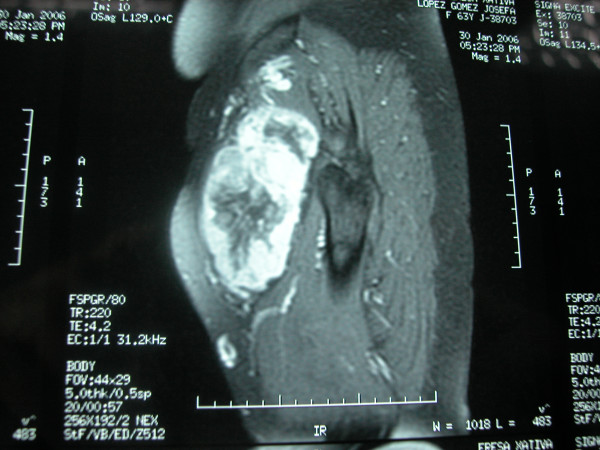
**Magnetic Resonance Imaging**. Tumour measuring 11 × 7 × 7 cm. in the left quadriceps.

**Figure 2:**
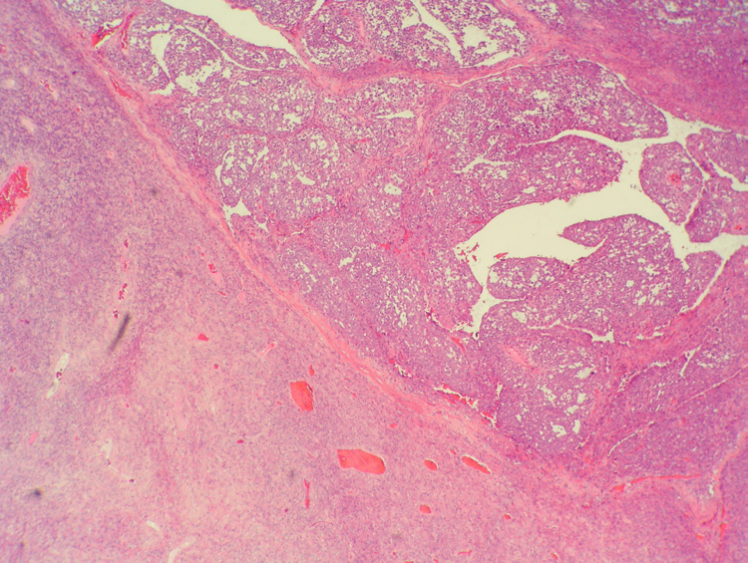
**Microscopic study, low magnification**. Epithelioid nodules near fusocellular areas H&E, 40×.

**Figure 3:**
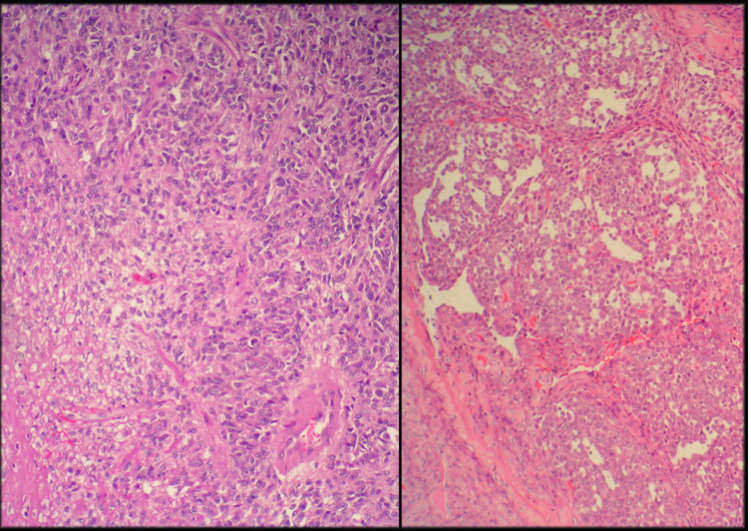
**Detail of the epithelioid and fusocellular areas**. Biphasic pattern, with epithelioid and fusocellular areas H&E, 100×.

**Figure 4:**
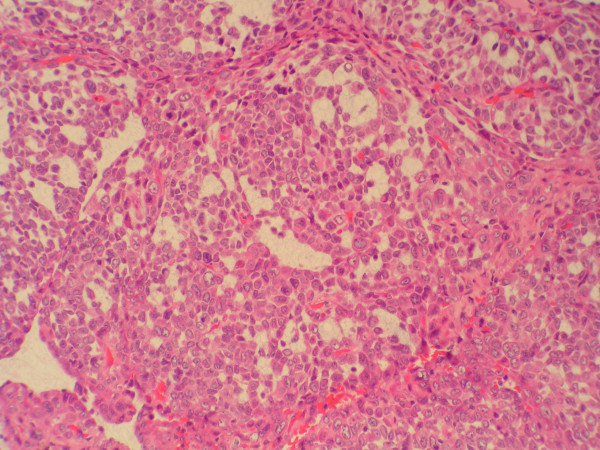
**Epithelioid cells, high magnification**. Round. polygonal cells, with eosinophilic cytoplasm and scarce mitosis, H&E, 200×.

**Figure 5:**
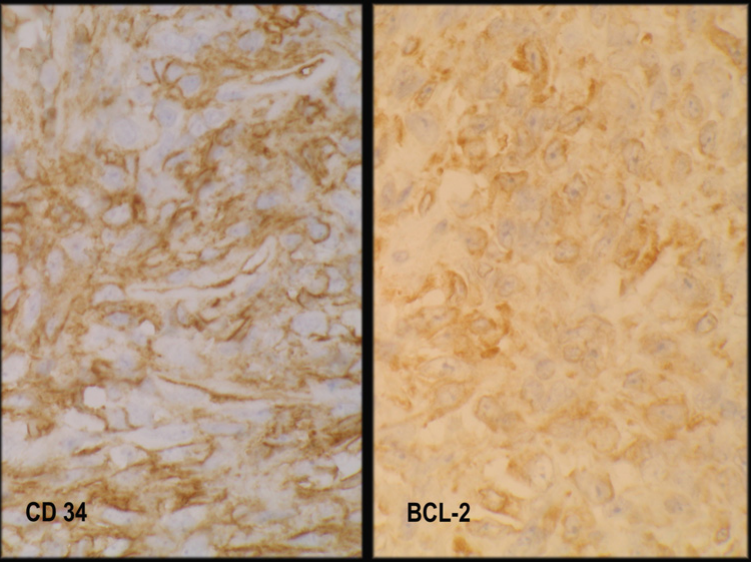
**Immunohistochemical results**. CD34 and bcl-2 immunostainig in epithelioid cells.
